# Suspected Radiation-Induced Complete Atrioventricular Block Presenting 39 Years After Childhood Thoracic Cancer Treatment: A Case Report

**DOI:** 10.7759/cureus.110116

**Published:** 2026-06-02

**Authors:** Carisa O Jimenez Chaparro, Carlos R Hernandez Nieves, Christian O Camacho Ramirez, Jose A Roman Ramos, Miguel A Echevarria Quintana

**Affiliations:** 1 School of Medicine, Universidad Autónoma de Guadalajara, Mayagüez Medical Center, Mayagüez, USA; 2 Internal Medicine, Centro Medico Episcopal San Lucas, Ponce, USA; 3 Cardiology, Centro Medico Episcopal San Lucas, Ponce, USA

**Keywords:** cardiac conduction abnormalities, long latency period, myocardial infarction type 2, primary lung malignancy, radiation-induced heart disease, suspected radiation-induced heart disease, third-degree atrioventricular block, thoracic radiation

## Abstract

Complete atrioventricular block is a serious cardiac conduction abnormality with multiple etiologies. We present the case of a 50-year-old man who presented to the emergency room with bradycardia found at home, associated with fatigue and exertional dyspnea, which eventually progressed from sinus rhythm to complete atrioventricular block with mild elevation in high-sensitivity troponin. Further inquiry revealed a remote history of childhood lung malignancy treated with surgical resection, chemotherapy, and thoracic radiotherapy approximately 39 years prior, representing one of the longest reported latency periods between thoracic radiation exposure and suspected radiation-induced complete atrioventricular block. The patient underwent permanent pacemaker placement with a favorable clinical outcome. This case report highlights the importance of detailed past medical history in identifying delayed cardiovascular complications of childhood cancer treatment. Clinicians should maintain a high index of suspicion when assessing conduction abnormalities as possible radiation-induced cardiac disease in cancer survivors, even decades after initial exposure.

## Introduction

Complete atrioventricular (AV) block, also referred to as third-degree block, is characterized by the absence of conduction of atrial impulses to the ventricles, resulting in AV dissociation [1]. Common etiologies include age-related degeneration of the conduction system, acute myocardial infarction, medication effects, infections, cardiac surgery, and congenital heart disease [1].

With improving survival rates among childhood cancer patients, the long-term cardiovascular consequences of cancer-directed therapies have become an increasingly recognized concern in clinical practice [2,3]. Conduction abnormalities represent a less recognized but clinically significant late complication of thoracic radiation therapy. Radiation-induced heart disease can affect multiple cardiac structures, including the coronary arteries, cardiac valves, pericardium, myocardium, and conduction system.

Latency periods between thoracic radiation exposure and clinically apparent conduction disease have been reported to range from several years to over two decades, with some isolated cases appearing beyond 20 years after initial exposure [2,3,4]. Radiation doses exceeding 35-40 Gy to the mediastinum, particularly with extended anterior-posterior fields commonly used during the 1980s, have been associated with increased risk of long-term cardiac injury, including conduction system fibrosis. Higher cumulative mediastinal doses confer progressively greater cardiac toxicity [5,6]. Thoracic radiation for pediatric lung malignancies during the 1980s typically employed extended treatment fields with generous margins that often included mediastinal structures, placing the AV node and surrounding conduction tissue at significant risk of incidental exposure [5,6]. Mediastinal radiation, in particular, has been associated with progressive fibrosis of the conduction system, with complete AV block representing one of its most severe late manifestations [4,5,6]. Notably, this case may represent one of the longest reported latency periods between thoracic radiation exposure and the development of suspected radiation-induced complete AV block.

We report the case of a middle-aged male patient with third-degree AV block in whom no apparent acute etiology was identified. A remote history of childhood lung malignancy treated with surgical resection and chemoradiation approximately 39 years prior served as a pivotal diagnostic consideration. Primary lung tumors in children are exceedingly rare, accounting for less than 0.2% of all pediatric malignancies [7,8]. Pulmonary metastases from extrathoracic pediatric malignancies such as Wilms tumor and sarcomas also represent an important consideration in this age group [9,10]. Regardless of histology, thoracic and mediastinal radiation was historically employed as part of multimodal therapy for pediatric lung malignancies, whether primary or metastatic, particularly during an era when treatment fields were less selective [9,10]. The development of complete AV block in this context is consistent with suspected radiation-induced conduction system injury. This case illustrates that such complications may manifest well beyond commonly reported latency periods, underscoring that a thorough past medical history remains an indispensable diagnostic tool in patients presenting with unexplained conduction abnormalities. Informed consent was obtained from the patient for publication.

## Case presentation

A 50-year-old man with a past medical history of hypertension and type 2 diabetes mellitus presented to the emergency department with a 48-hour history of fatigue and exertional dyspnea. He reported persistent bradycardia at home, with heart rates ranging from 42 to 55 beats per minute (bpm). The patient denied chest pain, syncope, or palpitations. He explicitly denied use of medications known to cause bradycardia, including beta-blockers, calcium channel blockers, and digoxin, and reported no recent infections.

On presentation, vital signs were notable for bradycardia (50 bpm), with otherwise stable hemodynamics. Physical examination was unremarkable aside from a decreased heart rate.

The initial electrocardiogram (Figure 1) demonstrated sinus rhythm at 60 beats per minute with right bundle branch block morphology. The PR interval was within normal limits at 160 ms (reference range 120-200 ms). The QRS complex was prolonged at 200 ms (reference range 80-120 ms), with an RSR′ pattern in lead V1 and broad, slurred S waves in the lateral leads, consistent with right bundle branch block. The heart rate of 60 bpm recorded on the initial electrocardiogram likely reflects spontaneous rate fluctuation, as the patient had reported home heart rates of 42-55 beats per minute in the preceding 48 hours and presented to the emergency department with a heart rate of 50 bpm on initial vital signs.

**Figure 1:**
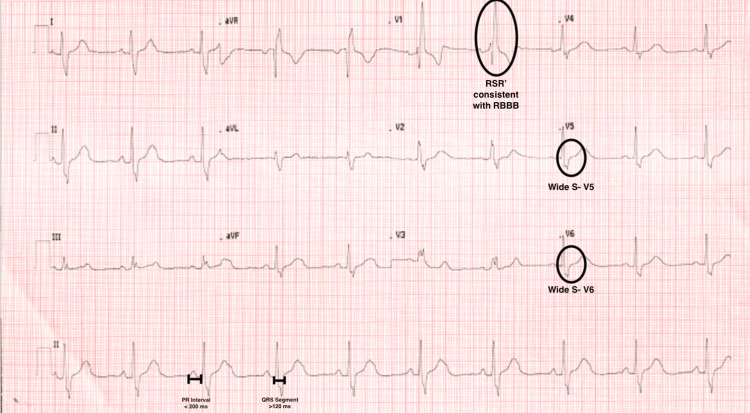
First electrocardiogram upon evaluation at the emergency department: sinus rhythm with right bundle branch block at 60 bpm.

Subsequent laboratory evaluation revealed mildly elevated high-sensitivity troponin levels, with an initial value of 58.43 ng/L and a repeat value of 59.30 ng/L (reference range 0-54 ng/L), without significant dynamic change. This stable pattern was interpreted as being more consistent with myocardial injury secondary to demand ischemia in the setting of profound bradycardia rather than acute coronary occlusion [11].

The patient was admitted to the internal medicine service. Given the troponin elevation and initial concern for a possible non-ST-elevation myocardial infarction, he was empirically managed with dual antiplatelet therapy, anticoagulation, and high-intensity statin therapy. Coronary angiography was deferred in the absence of ischemic ST-segment changes, anginal symptoms, and a non-dynamic troponin trend, collectively lowering the suspicion for acute plaque rupture.

Despite initial medical management, the patient subsequently developed progressive bradycardia with evidence of worsening AV conduction disease. A repeat electrocardiogram (Figure 2) demonstrated complete AV dissociation at a ventricular rate of 42 beats per minute, with P waves occurring independently of QRS complexes and no consistent PR interval relationship, consistent with third-degree AV block.

**Figure 2:**
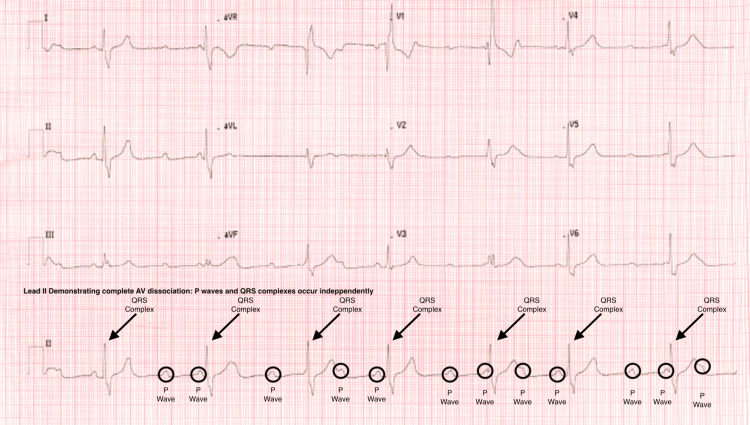
Electrocardiogram following acute coronary syndrome therapy, demonstrating complete atrioventricular (AV) block with AV dissociation, with no consistent relationship between P waves and QRS complexes at 42 bpm.

Given the development of complete heart block with potential hemodynamic compromise, the patient was transferred to the intensive care unit on the second day of admission, where a temporary transvenous pacemaker was placed via the right internal jugular vein.

A transthoracic echocardiogram revealed preserved left ventricular systolic function (ejection fraction >55%), mild aortic regurgitation, trace mitral regurgitation, and no pericardial effusion, findings that argued against an active pericardial or myocardial injury. The study was limited due to a poor acoustic window.

A permanent dual-chamber pacemaker was implanted the following day without complications. Post-procedural electrocardiogram (Figure 3) demonstrated a ventricular paced rhythm with broad QRS complexes demonstrating left bundle branch block morphology, consistent with right ventricular septal pacing and appropriate pacemaker function in the setting of persistent complete AV block, with AV dissociation and no measurable PR interval.

**Figure 3:**
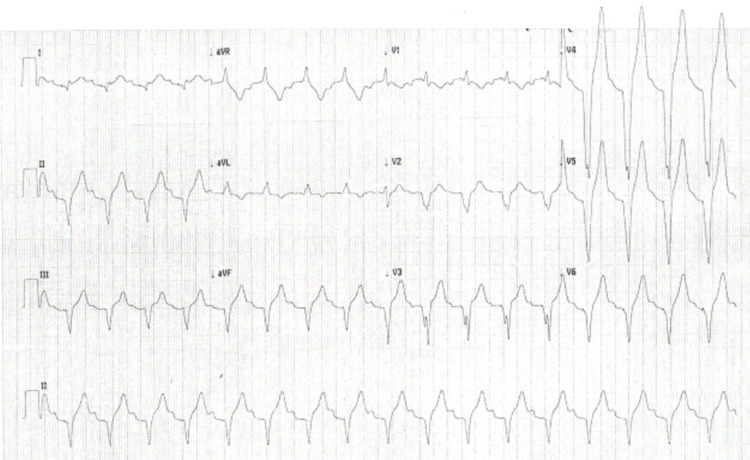
Electrocardiogram after permanent pacemaker placement, ventricular paced rhythm at 102 bpm, consistent with appropriate pacemaker function in the setting of complete AV block.

A chest X-ray performed after temporary transvenous pacemaker demonstrated right lower lobe linear atelectasis versus scarring, diffuse right hemithorax volume loss, and fibrotic changes in the right middle lobe (Figure 4). These chronic structural findings prompted further evaluation with chest CT, which confirmed extensive volume loss and fibrosis throughout the right lung field with fibrotic changes involving the right upper lobe extending to the apex and toward the mediastinum in proximity to central cardiac structures (Figure 5). Upon further questioning, the patient recalled a remote history of right lung malignancy at age 11, treated with surgical resection, chemotherapy, and thoracic radiotherapy. Notably, pleural thickening with calcification identified in the right lower lobe was deemed consistent with post-radiation pleuropulmonary sequelae in this context; no occupational or environmental asbestos exposure was identified upon directed questioning. Specific oncologic details, including tumor histology, radiation dose, field, and technique, could not be ascertained, as childhood treatment records were unavailable and the patient was unable to recall these specifics; this represents an inherent and acknowledged limitation of the present report.

**Figure 4:**
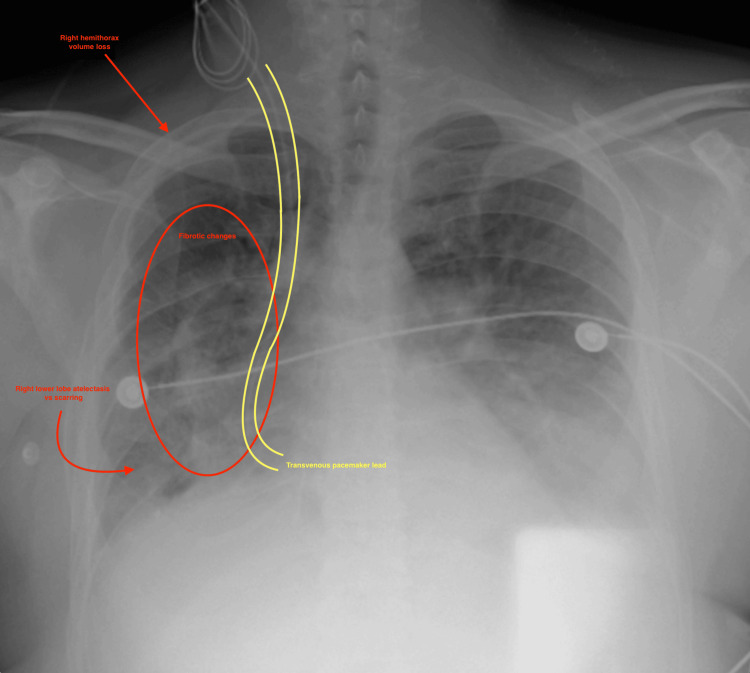
Chest X-ray demonstrating right lower lobe atelectasis versus scarring, diffuse right hemithorax volume loss, and fibrotic changes of the right middle lobe.

**Figure 5:**
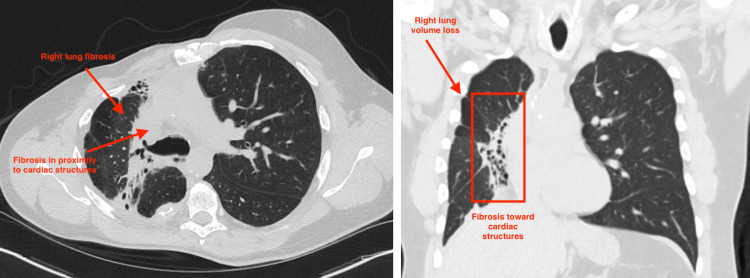
Axial and coronal view of chest CT demonstrating right lung volume loss with fibrotic changes involving the right upper lobe extending to the apex and in proximity to central cardiac structures.

No other reversible causes of AV block were identified; reversible and secondary etiologies, including but not limited to drug-induced causes, metabolic and endocrine disorders, infectious, autoimmune, infiltrative, ischemic, vagally mediated, and congenital or genetic causes, were systematically considered consistent with the 2018 ACC/AHA/HRS Guideline on the Evaluation and Management of Patients with Bradycardia and Cardiac Conduction Delay [1]. Drug-induced AV block was excluded, as the patient explicitly denied use of beta-blockers, non-dihydropyridine calcium channel blockers, digoxin, or other antiarrhythmic agents. Metabolic and endocrine causes were discarded based on laboratory findings, including a normal serum potassium of 4.5 mmol/L (reference range 3.5-5.1 mmol/L), sodium of 138 mmol/L (reference range 136-145 mmol/L), calcium of 9.2 mg/dL (reference range 8.3-10.6 mg/dL), and thyroid-stimulating hormone of 1.625 mIU/L (reference range 0.550-4.780 mIU/L). Among infectious etiologies considered, Lyme carditis was ruled out given the patient's residence in Puerto Rico, where *Borrelia burgdorferi* is not endemic, and his denial of travel to endemic regions or tick exposure. Other infectious etiologies, including bacterial endocarditis and acute rheumatic fever, were considered unlikely given the absence of fever, new murmurs, or systemic symptoms and a normal erythrocyte sedimentation rate (ESR) of 15 mm/hr (reference range 0-20 mm/hr). Autoimmune and inflammatory etiologies were considered unlikely, as no history of systemic autoimmune disease or systemic inflammatory symptoms was identified, and the normal ESR argued against an active inflammatory process. Cardiac sarcoidosis was considered unlikely, as echocardiography did not demonstrate basal septal thinning, regional wall motion abnormalities, or reduced ejection fraction, and serum calcium was normal at 9.2 mg/dL (reference range 8.3-10.6 mg/dL). Cardiac amyloidosis was considered unlikely given normal left ventricular wall thickness and the absence of a low-voltage QRS pattern on electrocardiography. Regarding ischemic causes, high-sensitivity troponin was mildly and non-dynamically elevated (58.43 and 59.30 ng/L; reference range 0-54 ng/L), a pattern interpreted as demand ischemia in the setting of profound bradycardia rather than acute coronary occlusion; serial electrocardiograms demonstrated no ST-segment changes, and the patient denied chest pain or anginal symptoms. Vagally mediated AV block was considered unlikely given the persistent nature of the conduction disturbance, with continuous AV dissociation documented on telemetry prior to pacemaker placement. A congenital or genetic etiology was considered unlikely given the absence of a family history of arrhythmia, sudden cardiac death, or pacemaker implantation, and the presence of sinus rhythm with AV conduction on the initial electrocardiogram at presentation. After exclusion of alternative etiologies, suspected radiation-induced conduction system injury was identified as the most likely etiology, given the patient's remote history of childhood thoracic radiotherapy. The patient tolerated the procedure well and was discharged in stable condition two days after permanent pacemaker implantation, with appropriate follow-up arranged. The clinical timeline is summarized in Figure 6.

**Figure 6:**
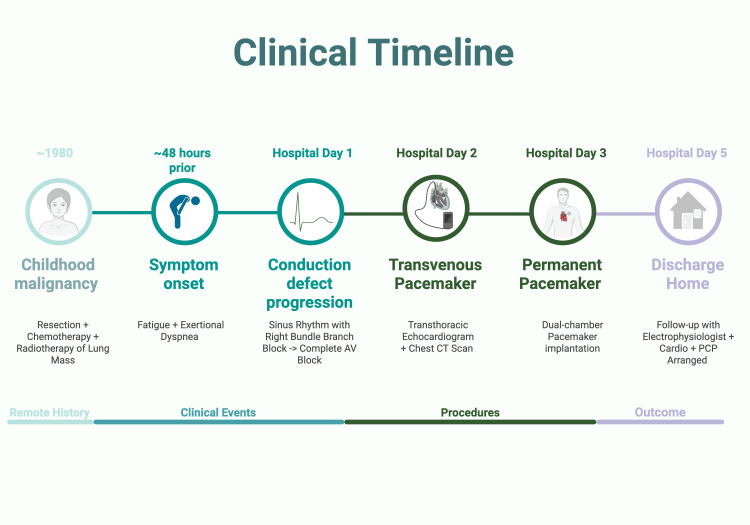
Clinical timeline of suspected complete AV block following childhood thoracic radiation. Clinical timeline summarizing the patient's presentation, diagnostic evaluation, and management. Created in BioRender (Toronto, Canada). Jiménez, C. (2026) https://BioRender.com/a68jr6d

## Discussion

Radiation therapy is an effective treatment for thoracic malignancies; however, it is associated with long-term cardiovascular complications known as radiation-induced heart disease [2,3]. These complications include coronary artery disease, valvular disease, pericardial disease, myocardial fibrosis, and, less commonly, conduction system abnormalities [2,3]. Studies have demonstrated that up to 75% of long-term survivors who received thoracic radiation have conduction defects on electrocardiogram, encompassing a spectrum from infra-nodal and bundle branch blocks to ectopy and sustained arrhythmias, with right bundle branch block being the most common given the anterior location of the right bundle branch [12]. This progression is exemplified in the present case, in which right bundle branch block preceded and ultimately advanced to complete AV block.

Radiation-induced conduction abnormalities are thought to result from progressive fibrosis of the AV node and surrounding conduction pathways [11]. Ionizing radiation damages the microvasculature supplying the conduction system, triggering a chronic inflammatory response that ultimately results in collagen deposition and fibrotic replacement of the specialized conductive tissue, a process that may continue progressing silently for decades after initial exposure [13]. Several case reports have described complete AV block as a late complication of mediastinal radiation therapy, supporting the association between prior radiation exposure and delayed conduction system disease [4,14,15,16]. Reported latency periods between radiation exposure and clinical manifestation typically range from one to over 20 years, with a mean of approximately 12 years in the largest case series [2,3,4]. In this patient, the development of complete AV block nearly four decades after exposure represents one of the longest latency periods reported in the literature.

Although the specific histology of this patient's childhood lung malignancy could not be determined, the cardiac consequences of the prior thoracic radiation exposure remain clinically significant. Whether the tumor was a primary lung malignancy, such as pleuropulmonary blastoma or carcinoid tumor, or a pulmonary metastasis from an extrathoracic malignancy, such as Wilms tumor or sarcoma, thoracic and mediastinal radiation was a common component of multimodal therapy for pediatric lung tumors during the 1980s [7,8,9,10]. Critically, radiation techniques of that era employed extended treatment fields with generous margins that routinely encompassed mediastinal structures, in part because advanced cross-sectional imaging was not yet widely available to precisely delineate tumor boundaries [5,6]. This placed the AV node and surrounding conduction tissue at significant risk of incidental exposure, consistent with the pattern of suspected radiation-induced injury observed in this patient [5,6].

Slama et al. proposed etiologic criteria to attribute complete AV block to prior radiation therapy, particularly in patients presenting at an age where the causal relationship may not be immediately apparent [4]. These criteria include a radiation dose exceeding 4,000 rads (40 Gy) to the cardiac area; a latency period of 10 years or more; abnormal interval electrocardiograms demonstrating bundle branch block; pericardial involvement; and associated cardiac or mediastinal radiation-induced lesions [4]. In the present case, three of these five criteria are definitively satisfied: (1) a latency period of 39 years far exceeding the minimum threshold; (2) progressive conduction disease evidenced by a right bundle branch block preceding complete AV block; and (3) associated radiation-induced lesions, evidenced by pleural thickening with calcification on CT consistent with post-radiation pleuropulmonary sequelae, as well as valvular lesions including mild aortic regurgitation and trace mitral regurgitation, both of which represent recognized manifestations of radiation-associated cardiac injury and fall under this criterion [4]. Orzan et al. specifically demonstrated that mild-to-moderate aortic and mitral regurgitation are present in virtually all patients with radiation-induced complete heart block and noted that otherwise unexplained valvular involvement lends important support to the diagnosis; even mild aortic insufficiency may represent a subtle clinical clue to radiation-induced damage of the aortic root [17]. Pericardial involvement could not be formally assessed given the limitations of the echocardiographic study secondary to a poor acoustic window, representing an indeterminate rather than absent criterion. The radiation dose criterion could not be assessed due to unavailable childhood treatment records; however, Slama et al. themselves acknowledged that the exact dose delivered to the heart is often difficult to ascertain because of treatment delays, multiple radiation fields, and changing protocols [4]. Overall, fulfillment of three Slama criteria, together with supportive valvular findings, strengthens the presumptive diagnosis of suspected radiation-induced conduction system disease in this patient.

In this case, the presence of mild elevation in high-sensitivity troponin without significant dynamic change suggests myocardial injury, likely, secondary to demand ischemia in the setting of severe bradycardia rather than primary coronary occlusion [11]. This distinction is clinically relevant, as it may help avoid unnecessary invasive interventions and supports appropriate management focused on treating the underlying conduction abnormality. Orzan et al. demonstrated significant coronary arterial stenoses, including ostial involvement, in patients with radiation-induced complete heart block, highlighting that coronary artery disease may coexist in this population [17].

The absence of cardiac MRI or electrophysiologic studies confirming conduction system fibrosis, combined with unavailable oncologic treatment records including radiation dose, field, and technique, precludes definitive causal attribution. The association between prior thoracic radiation and the development of complete AV block in this case therefore remains presumptive. It is acknowledged that incomplete historical records represent a well-recognized challenge in long-term cancer survivorship; large cohort studies have documented that many childhood cancer survivors treated decades ago lack detailed treatment records, and the CARE guidelines for case report writing emphasize transparency regarding what is known and unknown rather than requiring completeness of all historical data [18,19]. Without quantitative radiation data, the dose-response relationship cannot be established for this individual patient; however, the temporal association, fulfillment of three of five Slama diagnostic criteria, with pericardial involvement indeterminate and valvular lesions providing additional supportive evidence, along with systematic exclusion of alternative etiologies and imaging evidence of mediastinal fibrosis in proximity to central cardiac structures, collectively support suspected radiation-induced conduction system disease as the most likely explanation. Cardiac MRI was not performed during the hospitalization; future evaluation with cardiac MRI to assess for myocardial fibrosis patterns consistent with radiation injury is recommended as part of ongoing survivorship surveillance, contingent upon confirmation of MRI compatibility of the implanted device system [12].

Based on current cardio-oncology guidelines, childhood cancer survivors who received thoracic radiation should undergo baseline cardiovascular evaluation, including ECG and echocardiography, at the time of transition to adult care, with periodic reassessment every three to five years or sooner if symptoms arise [3,12]. Non-invasive coronary artery evaluation, including coronary CT angiography or coronary artery calcium scoring, is recommended beginning five years after radiation exposure in high-risk patients [3,6,19]. This case underscores the critical importance of lifelong cardiovascular surveillance in this population, as clinically significant conduction disease may emerge decades beyond the intervals typically anticipated.

## Conclusions

This case illustrates a suspected radiation-induced complete AV block presenting 39 years after childhood thoracic radiation therapy, with the diagnosis supported by fulfillment of three of five Slama etiologic criteria and systematic exclusion of alternative causes. The 39-year latency period extends beyond commonly reported intervals and reinforces that suspected radiation-induced conduction system disease may manifest well beyond the timeframes typically anticipated. In patients presenting with unexplained conduction abnormalities, particularly when common etiologies are absent, a detailed past medical history, including prior malignancy and radiation exposure, is essential for timely diagnosis. This case underscores the importance of lifelong cardiovascular surveillance in childhood cancer survivors who received thoracic radiation, including periodic electrocardiography, echocardiography, and non-invasive coronary artery evaluation as recommended by current cardio-oncology guidelines.
